# Psychiatric Symptom Dimensions Are Associated With Dissociable Shifts in Metacognition but Not Task Performance

**DOI:** 10.1016/j.biopsych.2017.12.017

**Published:** 2018-09-15

**Authors:** Marion Rouault, Tricia Seow, Claire M. Gillan, Stephen M. Fleming

**Affiliations:** a Wellcome Trust Wellcome Centre for Human Neuroimaging, University College London, London, United Kingdom; b Max Planck UCL Centre for Computational Psychiatry and Ageing Research, University College London, London, United Kingdom; c School of Psychology, Trinity College Dublin, Dublin, Ireland

**Keywords:** Cognitive neuroscience, Computational psychiatry, Confidence, Decision making, Metacognition, Psychopathology

## Abstract

**Background:**

Distortions in metacognition—the ability to reflect on and control other cognitive processes—are thought to be characteristic of poor mental health. However, it remains unknown whether such shifts in self-evaluation are due to specific alterations in metacognition and/or a downstream consequence of changes in decision-making processes.

**Methods:**

Using perceptual decision making as a model system, we employed a computational psychiatry approach to relate parameters governing both decision formation and metacognitive evaluation to self-reported transdiagnostic symptom dimensions in a large general population sample (*N* = 995).

**Results:**

Variability in psychopathology was unrelated to either speed or accuracy of decision formation. In contrast, leveraging a dimensional approach, we revealed independent relationships between psychopathology and metacognition: a symptom dimension related to anxiety and depression was associated with lower confidence and heightened metacognitive efficiency, whereas a dimension characterizing compulsive behavior and intrusive thoughts was associated with higher confidence and lower metacognitive efficiency. Furthermore, we obtained a robust double dissociation—whereas psychiatric symptoms predicted changes in metacognition but not decision performance, age predicted changes in decision performance but not metacognition.

**Conclusions:**

Our findings indicate a specific and pervasive link between metacognition and mental health. Our study bridges a gap between an emerging neuroscience of decision making and an understanding of metacognitive alterations in psychopathology.

Theoretical models suggest that alterations in metacognition, an ability to reflect on and evaluate one’s behavior, are characteristic of poor mental health 1, 2. If this evaluation process is disrupted, diverse and subtle changes in behavior can ensue (3). For instance, pervasive low confidence in one’s abilities may become self-fulfilling 4, 5, whereas overconfidence and blunted metacognition may lead to risky decision making (6) and delusional beliefs 7, 8, 9. Notably, the level of confidence is a relatively stable feature of individuals’ judgments that generalizes across different tasks 10, 11, 12 and has an inherited component (13), suggesting that it may represent a trait-level predictor of psychopathology.

However, establishing a formal relationship between metacognition and psychopathology has remained elusive for at least two reasons. First, changes in processes supporting decision formation, metacognition, or both may plausibly lead to widespread behavioral alterations. It is increasingly appreciated that there is a two-way relationship between decision making and metacognitive evaluation. Performing better at a task leads to greater confidence 14, 15, and confidence estimates in turn shape and control choices 12, 16, thereby “setting the switches” for lower-level decision processes 17, 18. Therefore, to isolate changes in metacognitive processes, it is critical to identify and control for confounding changes in performance 19, 20.

Second, for some symptom clusters, one would paradoxically predict both underconfidence and overconfidence (21). For instance, in schizophrenia, one might expect the presence of positive symptoms, such as delusions, to be associated with overconfidence (8), whereas negative symptoms, such as apathy, might be associated with underconfidence (22). One possibility is that this apparent paradox reflects issues with our use of DSM diagnostic categories in psychiatric research, where there is growing consensus that diagnostic labels, such as schizophrenia, are unlikely to reflect unitary, biologically plausible, or informative markers of mental health 23, 24, 25. In response, a new field of so-called computational psychiatry is emerging, with the aim of relating core brain processes underpinning complex behavior to transdiagnostic features of significance for mental health 26, 27, 28.

In the present study, we adopt a computational psychiatry approach, leveraging a large-scale general population sample (*N* = 995) 29, 30 to interrogate the relationship between decision making, metacognition, and self-reported psychopathology. We dissected and quantified distinct aspects of decision formation and metacognition using sequential sampling and signal detection-theoretical models 14, 20, 31, 32 in a perceptual decision-making task (33). Critically, a dimensional analysis uncovered dissociable relationships between distinct aspects of psychopathology and metacognition in the absence of any links to decision formation. Subjects with greater anxious-depressive symptoms exhibited lower confidence and improved metacognition, whereas a symptom dimension characterized by compulsive behavior and intrusive thought (not predicted by any questionnaire score alone) was associated with overconfidence and blunted metacognition. Our findings indicate that studying metacognitive mechanisms will be fruitful in bridging a gap between a neuroscience of decision making and core underpinnings of psychopathology.

## Methods and Materials

### Participants

Data were collected online using Amazon’s Mechanical Turk (experiment 1: 663 participants, 18–75 years of age; experiment 2: 637 participants, 18–70 years of age). Beyond the symptom questionnaires, no information about psychiatric diagnosis or medication was recorded (Supplemental Figure S1). It remains possible that at the extremes of the spectrum, certain participants would qualify for a psychiatric diagnosis and therefore have a higher likelihood of being treated with psychotropic medication, but our focus here is on continuous variation in psychopathology in the general population. Participants provided consent in accordance with procedures approved by the University College London Research Ethics Committee (Project ID 1260/003). Subjects were paid a base sum of $4 plus a $2 bonus conditional on both above-chance task performance and passing a check question (Supplemental Methods).

### Perceptual Decision-Making Task

**Figure 1:**
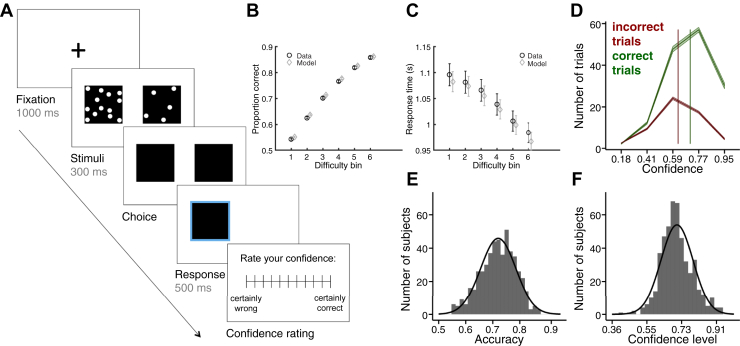
Decision-making task and behavior in experiment 1. **(A)** Perceptual decision-making task. Subjects were asked to judge which box contained the higher number of dots and to provide a confidence rating in each decision. Choice and confidence responses were unspeeded. **(B, C)** Behavioral data and drift-diffusion model fits. As difference in dots became greater, accuracy increased **(B)**, and response times decreased **(C)**. These features of the data were well captured by the drift-diffusion model. Error bars reflect SEM. **(D)** Average confidence rating distributions for correct and incorrect trials. Subjects gave higher confidence ratings for correct (green) than incorrect (red) trials. Shaded areas denote SEM; vertical lines denote the average confidence level for each response class. **(E, F)** Distributions of mean choice accuracy **(E)** and confidence level **(F)** across subjects (*n* = 498).

Participants were asked to judge which of two boxes contained the higher number of dots (Figure 1A) and to report their confidence in each judgment on a rating scale. Across both experiments, participants performed 210 trials divided into five blocks. In experiment 2, we added a calibration procedure to maintain a constant level of performance both during the experiment and across participants 19, 34. Further details are provided in the Supplement.

### Self-report Psychiatric Questionnaires

After the task, subjects completed standard self-report questionnaires assessing a range of psychiatric symptoms (Supplemental Figure S1), including depression (Zung Self-Rating Depression Scale) (35), generalized anxiety (Generalized Anxiety Disorder 7-item scale) (36), schizotypy (Short Scales for Measuring Schizotypy) (37), impulsivity (Barratt Impulsiveness Scale 11) (38), obsessive-compulsive disorder (OCD) (Obsessive-Compulsive Inventory-Revised [OCI-R]) (39), and social anxiety (Liebowitz Social Anxiety Scale) (40), and a short IQ evaluation (International Cognitive Ability Resource) (41) (see Supplemental Methods). In experiment 2, we added eating disorders (Eating Attitudes Test), apathy (Apathy Evaluation Scale), and alcoholism (Alcohol Use Disorders Identification Test) questionnaires.

### Exclusion Criteria

To ensure data quality, several exclusion criteria were applied for both task comprehension and performance (see Supplemental Methods). Across both experiments, approximately 23% of participants were excluded from further analysis, leaving 498 participants for experiment 1 and 497 participants for experiment 2. Exclusion criteria were predefined and reflect standard guidelines for online data collection (42), and the overall exclusion rate was consistent with a recent meta-analysis, which found that between 3% and 37% of the sample is typically excluded in web-based experiments (43).

### Drift-Diffusion Model

Decision formation was characterized using the drift-diffusion model (DDM), which models two-choice decision making as a process of accumulating noisy evidence over time with a certain speed, or drift rate (*v*), until one of two decision boundaries are crossed (32). The model was fit to accuracy-coded data with four free parameters: nondecision time, decision threshold, baseline drift rate (*v*_0_), and effect of dots difference on drift rate (*v*_δ_). Mean posterior estimates were extracted for entry into subsequent regression analyses. Full details of the model and fitting procedure are provided in the Supplement.

### Linear Regressions

We conducted linear regressions to examine the relationship between psychiatric symptoms, age, and IQ and task-related variables (accuracy, DDM parameters, confidence level, and metacognitive efficiency). *Z* scores of all regressors were calculated to ensure comparability of regression coefficients. For details of regression equations, see the Supplement. The code and data to reproduce regression analyses are freely available at https://github.com/metacoglab/RouaultSeowGillanFleming.

### Quantifying Confidence Level (Bias) and Metacognitive Efficiency

In experiment 2, we leveraged signal detection theory to characterize the sensitivity of an observer’s confidence reports to correct or incorrect judgments (19). This approach posits a generative model of the confidence data and returns a parameter, meta-*d′*, that reflects an individual’s metacognitive sensitivity (14). Meta-*d′* can be compared with decision *d′* to provide a relative measure of metacognitive efficiency, log(meta-*d′/d′*), controlling for task performance. Confidence level is independent of metacognitive efficiency (20) and reflects the tendency to use higher or lower confidence ratings regardless of their fluctuation owing to performance (see Supplemental Methods).

### Factor Analysis

In experiment 2, we applied a factor analysis to obtain a parsimonious latent structure for explaining shared variance at the item level across questionnaires (Supplemental Figure S2). We selected the number of factors based on Cattell’s criterion (44), in which a sharp elbow indicates the point at which there is little benefit to retaining additional factors. Using the same battery of questionnaires, Gillan *et al.* (29) found that a model with three underlying factors (labeled anxious-depression [AD], compulsive behavior and intrusive thought [CIT], and social withdrawal [SW]) provided the best account of the covariance across individual questionnaire items. Our sample size in experiment 2 (*n* = 497) provides a relatively low subject-to-variable ratio for de novo factor analysis. To ensure that our obtained solution replicates previous results obtained with this questionnaire set (29), we therefore compared the correlation structure of item loadings between our current study and that of Gillan *et al.* (29), who had access to a substantially higher subject-to-variable ratio (*N* = 1413 subjects).

## Results

In experiment 1 (*n* = 498), participants first performed a perceptual decision-making task in which they were asked to judge which of two boxes contained a greater number of dots and to rate their confidence in each decision (Figure 1A). Next, they responded to a number of self-report questionnaires assessing a range of mental health symptoms, followed by a shortened IQ evaluation (see Supplemental Methods).

As expected, choice accuracy increased and response times decreased as the difference in number of dots became greater (Figure 1B, C). Across trials, reported confidence was reliably related to decision accuracy (median within-subject correlation: ρ = .25, *p* < .0005, ranging from ρ = −.05 to ρ = .59), owing to subjects’ reporting higher confidence ratings for correct than for incorrect choices (Figure 1D). Across participants, we observed considerable variability in both performance (Figure 1E) and confidence (Figure 1F); however, performance accounted for only 3.2% of the variance in confidence levels (between-subject correlation: ρ = .18, *p* < .0005). This allowed us to separately study the contribution of psychiatric symptoms to decision formation (speed and accuracy) and metacognition.

To further dissect processes underpinning decision formation, we fitted a DDM to participants’ choices and response times 31, 32. The DDM models two-choice decision making as a process of accumulating noisy evidence over time with a certain speed, or drift rate. Simulations of the fitted model show that it captured variation in both accuracy (Figure 1B) and response times (Figure 1C) as a function of difficulty. Consistent with previous studies (45), we found that age and IQ predicted changes in decision formation, with older age associated with slower, less accurate decisions (Supplemental Figure S6A and Supplemental Results). In contrast, neither age nor IQ was related to confidence (Supplemental Figure S6A).

**Figure 2:**
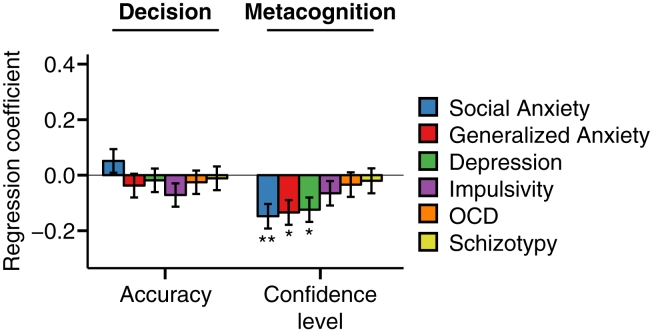
Association between decision (left) and metacognitive (right) variables with self-reported psychopathology in experiment 1 (*n* = 498). Each psychiatric symptom was examined in a separate regression, additionally controlling for the influence of age, gender, and IQ. The y axis indicates the change in each dependent variable for each change of 1 SD of symptom scores. Anxiety and depression symptoms were related to lower confidence level in the absence of a change in decision accuracy. Error bars denote SE. *p < .05, **p < .01, corrected for multiple comparisons over the number of dependent variables tested. See also Supplemental Figure S7A. OCD, obsessive-compulsive disorder.

We next turned to the relationship between decision making, metacognition, and psychiatric symptoms (self-reported social anxiety, generalized anxiety, depression, impulsivity, OCD, and schizotypy), systematically controlling for the effects of age, IQ, and gender (Figure 2 and Supplemental Figure S1). In contrast to age, psychiatric symptoms were not associated with decision accuracy (Figure 2) or DDM parameters governing decision formation (Supplemental Figure S7A). However, in line with our hypothesis, we found that self-reported depression, social anxiety, and generalized anxiety scores all were associated with lower confidence level (all β < −.12, all *p* < .05) (Figure 2). Impulsivity, OCD, and schizotypy scores exhibited no association with confidence level (all *p* > .05).

**Figure 3:**
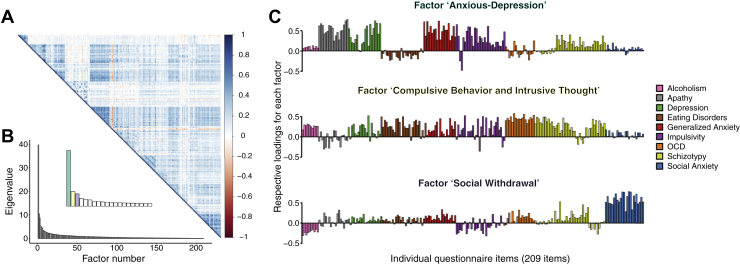
Three latent factors (dimensions) explained the shared variance between all questionnaire items. **(A)** Correlation matrix of 209 questionnaire items showing significant correlations between the answers to questionnaire items across subjects. The color scale indicates the correlation coefficient. **(B)** Eigenvalues from the factor analysis revealing a three-factor solution that best accounted for our data. We labeled these factors anxious-depression, compulsive behavior and intrusive thought, and social withdrawal, according to the strongest individual item loadings. The inset corresponds to a zoom on the first few factors. **(C)** Item loadings onto each factor, color-coded by questionnaire. See also Supplemental Figures S1 and S2. OCD, obsessive-compulsive disorder.

In keeping with good statistical practice in large datasets, we set out to replicate these effects in a second experiment (*n* = 497), while addressing two limitations of experiment 1. First, we observed strong correlations between individual questionnaire scores consistent with comorbidity between these constructs (e.g., generalized anxiety and depression correlated at ρ = .75). Moreover, even within a particular questionnaire, different items may map onto separable latent factors, which are unobservable in traditional analyses. The set of questionnaires in experiment 1 was not a priori designed to enable the identification of such latent factors. To address this issue, we included additional questionnaires allowing identification of underlying transdiagnostic psychiatric dimensions through application of factor analysis (29). We identified three dissociable factors (dimensions) that cut across the nine questionnaires from which the 209 items were drawn (Figure 3), replicating previous findings (Supplemental Figure S9). These factors were labeled AD, CIT, and SW (see Supplemental Methods for further details).

Second, to more precisely isolate shifts in metacognition from fluctuations in decision performance, we equated performance across individuals using a continuous staircase procedure (Supplemental Figure S4C) 19, 34. Importantly, in experiment 2, this design change allowed us to compute not only confidence level (metacognitive bias) but also metacognitive efficiency (meta-*d′/d′*). Confidence level indexes a general tendency to respond with higher or lower confidence ratings regardless of objective performance, whereas metacognitive efficiency quantifies how well one distinguishes between correct and error trials 10, 20; both measures were empirically dissociated in the current dataset (ρ = .036, *p* = .42).

**Figure 4:**
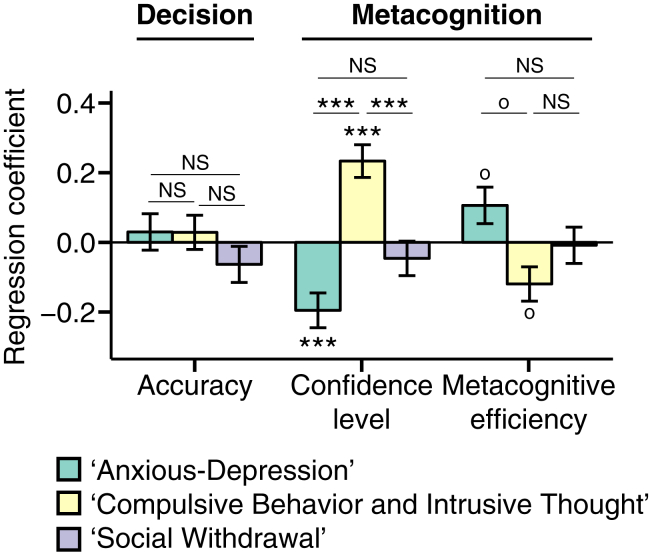
Factor analysis on the correlation matrix of 209 questionnaire items revealed a three-factor solution comprising anxious-depression, compulsive behavior and intrusive thought, and social withdrawal dimensions. Entry of these factors into a multiple regression model predicting decision formation and metacognition revealed bidirectional effects of anxious-depression and compulsive behavior and intrusive thought factors on confidence level, despite no relationships with performance. ^o^*p* < .05 uncorrected, ***p < .001 corrected for multiple comparisons over the number of dependent variables tested. See also Supplemental Figures S7C and S8. NS, not significant.

Consistent with experiment 1, we found no association between psychiatric symptoms and decision formation (Supplemental Figures S5 and S7B), despite replicating significant negative relationships with confidence level (apathy β = −.14, *p* < .01, generalized and social anxiety both β = −.10, *p* < .05 uncorrected) (Supplemental Figure S5). We next tested for an association between subjects’ scores on the three identified symptom dimensions and their separately measured profiles of decision formation and metacognition (Figure 4). When including all three factors in the same regression model (and controlling for IQ, age, and gender), accuracy and decision formation parameters exhibited no relationship with psychiatric factors (Figure 4 and Supplemental Figure S7C). However, the AD factor was significantly associated with lower confidence level (β = −.20, *p* < .001), whereas the CIT factor was significantly associated with higher confidence level (β = .23, *p* < .001) (Figure 4). Importantly, the identified subcategories of symptoms related to heightened confidence level were not visible in standard questionnaires (Figure 2 and Supplemental Figure S5)—highlighting the power of a dimensional analysis. Metacognitive efficiency (meta-*d′/d′*) exhibited the reverse relationship with symptom clusters: it was increased in subjects with higher scores on the AD factor and decreased in subjects with higher scores on the CIT factor (Figure 4) (note that these findings did not survive correction for multiple comparisons and should therefore be interpreted with caution) (AD, β = .11, *p*_uncorrected_ = .04; CIT, β = −.12, *p*_uncorrected_ = .02).

We next asked whether these positive and negative relationships between metacognition and symptom clusters were significantly different from one another. As expected, given their opposite signs, the coefficients for confidence level (*p* < .0001) and metacognitive efficiency (*p* = .03) differed between the AD and CIT factors. Confidence level coefficients additionally differed between CIT and SW (*p* < .0002) but not between AD and SW (*p* = .07). Metacognitive efficiency did not differ between SW and either AD or CIT (both *p* > .16). Notably, the absolute magnitudes of these effects were similar: confidence level was not more strongly associated with AD than with CIT (*p* = .5); likewise, metacognitive efficiency was not more strongly associated with AD than with CIT (*p* = .8). All relationships between symptom dimensions and metacognition remained when additionally controlling for all aspects of decision formation in the same regression model (Supplemental Figure S8). Finally, and importantly, our results could not be ascribed to a trivial anticorrelation between AD and CIT scores. While our factor analytic approach allowed factors to be correlated, we in fact found that AD and CIT were positively correlated (ρ = .36)—the opposite of what would be required to produce a spurious association with metacognition. Together, these results reveal that the AD and CIT symptom dimensions exert equal and opposite effects on two key aspects of metacognition—confidence level and metacognitive efficiency.

To assess the relative significance of these effects, we next entered metacognitive variables and accuracy as predictors of individual factor scores in separate regressions. Factor scores were significantly explained by confidence level (β = −.13 for AD and β = .15 for CIT, both *p* < .003) but not accuracy (both *p* > .6) or metacognitive efficiency (*p* = .1 for AD, trend at *p* = .04 for CIT). In addition, the association between each factor and confidence level effect was greater in magnitude than the corresponding relationship with accuracy (both *p* < .03).

**Figure 5:**
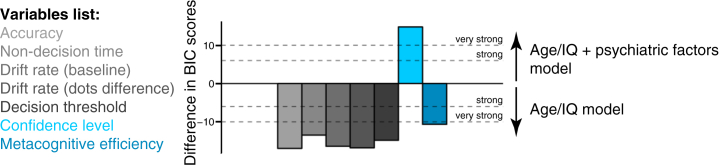
Model comparison. Taking into account both goodness of fit and parsimony, model comparison provided strong evidence for including psychiatric factors in addition to age and IQ for explaining confidence level. Age/IQ model: Variable ∼ age + IQ + gender. Age/IQ + psychiatric factors model: Variable ∼ anxious-depression factor score + compulsive behavior and intrusive thought factor score + social withdrawal factor score + age + IQ + gender. See also Supplemental Figure S6C. BIC, Bayesian information criterion.

To further quantify the extent to which including psychiatric factor scores explains individual differences in decision formation and metacognition, we computed the Bayesian information criterion for each regression model (Supplemental Methods). A simpler age/IQ–only model was able to account for decision formation and metacognitive efficiency (Figure 5). Indeed, it was notable that there was very strong evidence against the additional complexity induced by including psychiatric factors in models of decision formation. In contrast, and in keeping with our regression analyses, there was very strong evidence for including psychiatric symptom dimensions in addition to age and IQ to explain confidence level.

## Discussion

While distortions in self-evaluation are theorized to occur in many disorders of mental health, it has remained unknown whether these changes are due to selective alterations in metacognition and/or a downstream consequence of changes in sensory and decision processes. In this article, we show that self-reported psychiatric symptoms are associated with specific shifts in confidence but not performance in a controlled perceptual decision-making task. Our quantitative approach revealed two distinct relationships between psychopathology and metacognition: an AD symptom dimension was associated with lower confidence level and heightened metacognitive efficiency, and a CIT symptom dimension was associated with higher confidence level and disrupted metacognitive efficiency, despite accuracy and parameters governing decision formation remaining unaffected. This relationship was found across different methods of eliciting confidence: a numerical probability scale (0%–100% correct) in experiment 1 and a verbal scale (from guessing to certain) in experiment 2. Our findings suggest an endogenous set-point for confidence, in keeping with recent evidence that confidence level represents a stable individual difference that transcends both task and temporal focus 10, 46. Taken together, our findings reveal that shifts in metacognitive evaluation represent a specific and pervasive behavioral correlate of subclinical psychopathology.

A relationship between lower confidence level and an AD symptom dimension is consistent with depression’s being characterized by pervasive negative shifts in self-evaluation 5, 47, 48. For instance, patients with depression overattribute negative outcomes and underattribute positive outcomes to self-performance compared with control subjects (49). More broadly, dysfunctional self-evaluation may engender low self-efficacy, in which failures are attributed to low ability rather than to insufficient effort or external circumstances, in turn leading to negative beliefs about one’s ability to cope with difficulties and overcome challenges (3). Supporting this idea, theoretical simulations of an evolutionary model have shown that, counterintuitively, maintaining overconfidence can produce fitness benefits by promoting action (6). The strongest predictor of lowered confidence in experiment 2 was the apathy score, and brain regions involved in decision evaluation and motivation are also predictive of changes in apathy (50). It is therefore plausible that some symptoms of apathy and depression may emerge partly through a systematic undervaluation of one’s abilities 22, 51. Interestingly, the AD symptom dimension also showed a weaker positive relationship with metacognitive efficiency, consistent with greater insight into performance fluctuations.

In contrast, a symptom dimension characterizing CIT was associated with heightened confidence level and disrupted metacognition. Whereas this factor captures shared features of OCD, schizotypy, and eating disorders (29), critically, no individual questionnaire score significantly predicted heightened confidence. Only through identification of latent factors was a relationship between metacognition and CIT psychopathology uncovered. This finding has important ramifications for emerging reports of metacognitive alterations in psychiatric disorders. For instance, whereas some studies infer underconfidence in patients with OCD, manifest by repeated checking behaviors (52), other authors have observed that confidence in perceptual decision making is positively related to OCI-R scores (53). Conversely, Banca *et al.* (54) observed changes in decision formation parameters without changes in confidence levels in patients with OCD, albeit selectively on high difficulty trials. When examining raw OCI-R scores in our study, it is notable that we also find a trend-level increase in decision threshold in the absence of any effect of confidence. However, as OCD is often comorbid with anxiety, consistent with a subset of the OCI-R items positively loading on the AD factor in experiment 2 (Figure 3C), an anxiety-related component could explain previous observations of underconfidence in patients. Instead, our findings of disrupted metacognition in high-CIT individuals is consistent with recent findings of lowered metacognitive efficiency in high versus low compulsive participants who were matched for depression and anxiety symptoms (55). Such considerations underscore the relevance of applying a dimensional approach to relate cognitive differences to psychopathology (26).

Several items from the schizotypy questionnaire also contributed to the CIT construct and were found to be positive predictors of confidence level in our independent supervised analysis. Our results are therefore partly consistent with previous evidence of overconfidence and a jump-to-conclusions bias in patients with schizophrenia and schizoaffective disorders, i.e., disorders that map onto the CIT dimension 7, 8. However, we also found evidence against a corresponding relationship between schizotypy and parameters governing the process of decision formation. If overconfidence reflected a generalized bias in evidence accumulation, one would also expect it to affect task performance, for instance, through adjustments in the threshold amount of evidence needed to make a decision. Instead, our findings are of a strikingly specific link between psychopathology and metacognition. As we discuss below, it is possible that in other tasks, a mutual relationship between metacognition and decision making would manifest as a change in subsequent adjustment of first-order performance. Gillan *et al.* (29) found that a CIT symptom dimension was associated with a reduction in goal-directed control, potentially conferring vulnerability to developing rigid habits. Overconfidence could impair behavioral flexibility through formation and reinforcement of more rigid beliefs, which in turn would predict reductions in goal-directed control. Alternatively, confidence could be a distinct aspect of decision making that is altered in these individuals (56).

There is growing evidence that decision formation and metacognitive evaluation maintain a reciprocal relationship. Task performance influences confidence, and beliefs about self-efficacy determine the goals one chooses to pursue (3). Here we dissected decision formation and metacognition in a simple perceptual decision task that minimized requirements for learning, and thus from a normative point of view, confidence was less useful for behavioral adjustments. Indeed, this aspect of our experimental design was critical for isolating metacognitive shifts from changes in decision performance. In many other settings, accurately inferring one’s confidence in a task is an important indicator of whether a previous decision should be revised 17, 57, whether a subsequent step in a chain of decisions should be initiated (58), or more generally when it is advantageous to deliberate (59) or engage cognitive control (18). Our findings speak to computational models of confidence (15): while previous work has focused on modeling trial-level determinants of decision confidence 60, 61, the between-subjects variance typically captured by one or more free parameters in such models could reflect systematic trait-level differences among individuals. In turn, because widespread alterations in behavioral control are a pervasive characteristic of many mental disorders (26), our results suggest that alterations in metacognitive computation reflect a critical component of transdiagnostic psychopathology. It remains to be determined which step in the confidence computation is altered—for instance, alterations in the estimation of perceptual uncertainty, representations of self-action, and/or a mapping onto explicit reports could be affected. Answering this question may profit from novel tasks that enable disentangling elements of a confidence computation—for instance, selective changes in the influence of evidence variability, postdecisional processing, and/or action kinematics. Future work should also investigate how changes in metacognition impact cognitive control, learning, and behavioral adaptation, and determine how such control processes go awry in psychiatric disorders.

We stress that we did not screen for a categorical presence or absence of psychiatric disorders using structured clinical interviews; instead, we collected a general population sample with continuous variation along self-reported symptom dimensions (see Methods and Materials). As such, there are limits as to what we can infer about patients with psychiatric diagnoses from these data. However, prior work has shown that this methodology maps closely onto findings from small sample case-control designs. For example, failures in goal-directed (model-based) planning observed in patients with OCD that has been carefully diagnosed are mirrored in self-report scores in general population samples [patients with a diagnosis (62), general population sample (29)]. Furthermore, Rutledge *et al.* (63) found a comparable influence of expected values and reward prediction errors on momentary mood ratings in laboratory-based depressed and control participants compared with online participants with high and low depression scores (Beck Depression Inventory). The advantage of our methodology over more standard approaches is that a large sample allows us to control for, and indeed leverage, individual patterns of dimensional psychopathology on a within-participant basis—something that has not been possible in typical case-control studies. As such, this approach provides a powerful new pathway toward testing the merits of a dimensional view of psychiatry (25), consistent with the broader goals of the burgeoning computational psychiatry movement 26, 27.

In this article, we used perceptual decision making as a model system, allowing precise control over performance to reveal relationships between symptoms and metacognition. It remains to be explored how our findings may extend to other types of decisions (e.g., value-based) or other cognitive domains, such as memory. However, recent evidence points toward metacognition relying in part on domain-general resources, suggesting that findings from the present study are likely to generalize to other scenarios. For instance, there are shared neural and behavioral correlates of metacognition across visual, auditory, and tactile modalities (64) and between perception and memory (65). Moreover, a recent study of older participants found that metacognition in a go/no-go task correlated with monitoring deficits in daily life (66). In turn, confidence level and metacognitive efficiency have been linked to different adaptive benefits. On one hand, well-calibrated beliefs about performance (high metacognitive efficiency) may facilitate control of behavior, for instance, by modulating resource allocation and exploration (18) and cognitive offloading (67). On the other hand, appropriate bias/confidence level is linked to self-efficacy and educational achievement 3, 68, whereas excessive confidence may lead to maladaptive risk taking (69). It is hoped that our findings on metacognition may hold implications for treatment development: beliefs about one’s abilities represent a promising target for therapy in anxiety and depression (2). Furthermore, animal models now exist for understanding confidence computation at the level of neural circuits in both rodents and nonhuman primates (70). Understanding the mechanisms supporting metacognition may allow development of behavioral and neural interventions to restore accurate self-evaluation in the future (71). For instance, providing false feedback to healthy individuals engaged in a perceptual decision-making task is sufficient to boost confidence and self-efficacy and heighten subsequent task performance (4). Thus, by applying a transdiagnostic approach to the quantification of decision making and metacognition, strategies for ameliorating evaluative deficits in psychiatric disorders may be uncovered.
