# Supplementary material for: Psychiatric Symptom Dimensions Are Associated With Dissociable Shifts in Metacognition but Not Task Performance
Source: Biol Psychiatry. 2018 Sep 15;84(6):443–51. doi: 10.1016/j.biopsych.2017.12.017 (PMC6117452; doi:10.1016/j.biopsych.2017.12.017)
Supplement: Supplemental Material [file mmc1.pdf]

Psychiatric Symptom Dimensions Are Associated With Dissociable Shifts in Metacognition But Not Task Performance

Supplemental Information

Supplemental Methods

Behavioral Task

Experiment 1

We employed a perceptual decision-making task together with trial-by-trial confidence ratings (1) (Figure 1A). On each trial, participants were first presented with a fixation cross for 1000 ms. Two black boxes filled with differing numbers of randomly positioned white dots were then presented for 300 ms. One box was always half-filled (313 dots out of 625 positions), while the other box contained an increment of +1 to +70 dots compared to the standard. After 300 ms, the dots disappeared, leaving the black boxes on screen until a keyboard button press response was made. Participants were asked to judge which box had the highest number of dots. The left/right position of the target box was pseudo-randomised across all trials and within each of 5 difficulty bins. The chosen box was highlighted for 500 ms. On every trial, subjects were asked to report their confidence in their response on a rating scale. In Experiment 1, we used a full 11-point probabilistic rating scale (1=certainly wrong, 3=probably wrong, 5=maybe wrong, 7=maybe correct, 9=probably correct, 11=certainly correct) (2). No feedback was provided during the task. Participants performed 210 trials, in 5 blocks. In addition, before starting the experiment, participants were required to select on an 11-point scale their global expected performance level in the task relative to others (*global pre-task confidence*) (ranging from 1=1st percentile (worst) to 11=100th percentile (best)), together with a maximum and minimum expected performance level. After completing the task, participants were again asked to rate their expected performance level in the task relative to others, using the same scale (*global post-task confidence*). The entire experiment was coded in JavaScript with JsPsych version 4.3 (3).

Experiment 2

Experiment 2 was similar to Experiment 1 with the following exceptions. We used a 6-point scale for confidence with verbal rather than numerical labels (from 1=guessing to 6=certainly correct) in order to establish that our results were not specific to the scale type used. We also added a calibration procedure to maintain a constant level of performance during the experiment and across participants (4; 5). We implemented a two-down one-up staircase procedure with equal step-sizes for steps up and down. The step-size was calculated in log-space, with a starting point of 4.2 (+70 dots), changing by ± 0.4 for the first 5 trials, ± 0.2 for the next 5 trials and ± 0.1 for the rest of the task. The staircase was initiated during the 25 practice trials to minimize burn-in period. In addition, pre- and post-task global confidence ratings were omitted to avoid possible biasing of subsequent trial-by-trial confidence ratings.

Self-report Psychiatric Questionnaires

Experiment 1

After performing the behavioral task, subjects completed questionnaires assessing a range of psychiatric symptoms (Figure S1). Six self-report questionnaires were administered:

- *Depression* using the Self-Rating Depression Scale (SDS) (6),
- *Generalised anxiety* using the Generalised Anxiety Disorder 7-Item Scale (GAD-7) (7),
- *Schizotypy* using the Short Scales for Measuring Schizotypy (SSMS) (8),
- *Impulsivity* using the Barratt Impulsiveness Scale (BIS-11) (9),
- *Obsessive Compulsive Disorder* (OCD) using the Obsessive-Compulsive Inventory–Revised (OCI-R) (10), and
- *Social anxiety* using the Liebowitz Social Anxiety Scale (LSAS) (11).

Lastly, a proxy for IQ was collected using the International Cognitive Ability Resource (I-CAR) sample test which includes 4 item types of three-dimensional rotation, letter and number series, matrix reasoning and verbal reasoning (16 items total) (12).

Experiment 2

We expanded our battery of clinical questionnaires to allow us to probe the generalizability and specificity of our effect on confidence. Specifically, using a large battery of questionnaires (described below), Gillan et al. (2016) found that a model with three underlying factors (Anxious-Depression, Compulsive Behavior and Intrusive Thought, and Social Withdrawal) provided a good account of the covariance across individual questionnaire items. To enable application of this method, the questionnaires from Experiment 1 were modified as follows. The depression (SDS), schizotypy (SSMS), impulsivity (BIS-11), OCD (OCI-R), social anxiety (LSAS) and IQ (I-CAR) tests remained unchanged. The following changes were made:

- *Generalised Anxiety* questionnaire was replaced by the State Trait Anxiety Inventory (STAI) Form Y-2 (13),
- *Alcoholism* was assessed with the Alcohol Use Disorders Identification Test (AUDIT) (14),
- *Apathy* was assessed with the Apathy Evaluation Scale (AES) (15), and
- *Eating disorders* was assessed with the Eating Attitudes Test (EAT-26) (16).

The order of questionnaire administration was fully randomised.

Participants

Subjects were recruited from Amazon's Mechanical Turk. They were based in the USA, 95% of their previous jobs on Mechanical Turk were approved and all were ≥18 years old; subjects were otherwise anonymous. Subjects provided consent by clicking 'I confirm that I wish to continue' after reading the study information and consent pages online, in accordance with procedures approved by the UCL Research Ethics Committee.

Experiment 2. We conducted a power analysis to determine the appropriate sample size (17) from the correlation between the confidence residuals (controlling for age, IQ and gender) and depression scores in Experiment 1 (ρ=-0.129). To assess the relationship between confidence and depression scores with 80% power in Experiment 2, we required 470 subjects (after applying exclusion criteria, see below). We collected data from N=637 participants, of whom 318 were female (50%) with ages

ranging from 18 to 70 (mean=35.0, SD=10.5) years. As in Experiment 1, subjects were paid a base sum of $4 plus $2 bonus if their (calibrated) task performance was between 60-85% correct and they passed a “check” question.

Exclusion Criteria

The challenging nature of the perceptual decision-making task ensured that it was impossible to perform significantly above chance level if subjects were not paying careful and sustained attention to the stimuli. To additionally ensure data quality, several exclusion criteria were applied.

Experiment 1

Participants were required to pass all of the following 6 tests to be included:

A) Prior to the task, a comprehension test was administered regarding the use of the 11-point probabilistic confidence rating scale (1=certainly wrong, 3=probably wrong, 5=maybe wrong, 7=maybe correct, 9=probably correct, 11=certainly correct). They were asked where on the scale they should choose if 1) they were *sure* they made the *correct* judgement and 2) if they were *sure* they made a *mistake* in their judgement. Subjects failed the comprehension test if they answered less than 8 in response to (1) (n=88) and greater than 4 (n=34) in response to (2). In total, 90 (13.57%) subjects did not pass this criterion.

B) Participants were required to select on an 11-point scale their expected performance level in the task relative to others, together with maximum and minimum expected performance levels. Subjects passed this test if these ratings were transitive, i.e. minimum ≤ average ≤ maximum confidence. 65 (9.80%) subjects did not pass.

C) After completing the experimental task, participants were again asked to rate their expected performance level in the task relative to others; the same inclusion criterion was applied as in (B). 51 (7.69%) subjects did not pass.

D) Subjects with below- or near-chance task performance i.e. <55% correct were excluded (n=35, 5.28%).

E) Subjects were excluded if they incorrectly responded to a “catch” question: “If you are paying attention to these questions, please select ‘A little’ as your answer”. 20 (3.02%) subjects did not pass.

F) Subjects who always selected the same trial-by-trial confidence rating were excluded (n=2, 0.30%).

Combining all exclusion criteria, 165 (24.9%) participants were excluded, leaving N=498 participants for analysis. Our exclusion criteria were pre-defined and reflect standard guidelines for online data collection (18), and our exclusion rate was consistent with a recent meta-analysis who found that between 3% and 37% of the sample is typically excluded in online data collection (19).

Experiment 2

Participants from Experiment 1 were prohibited from taking part in Experiment 2. Subjects were required to pass the following tests to be included:

A) Prior to the experimental task, a comprehension test was administered regarding the use of the 6-point confidence rating scale (1=guessing, 6=certainly correct). They were asked where on the scale they should choose if 1) they were *very sure* they made the *correct* judgement and 2) if they were *very unsure* they made the *correct* judgement. We excluded subjects who failed the comprehension test if they answered less than 5 in response to (1) (n=89) and more than 2 in response to (2) (n=86). In total, 124 (19.47%) subjects did not pass this criterion.

B) Subjects who performed outside the range 60-85% correct were excluded (n=9, 1.41%).

C) As in Experiment 1, subjects who failed the “catch” question were excluded. 31 (4.87%) subjects did not pass.

D) Subjects who always selected the same trial-by-trial confidence rating were excluded (n=2, 0.31%).

In total, 140 (22.0%) participants were excluded, leaving N=497 participants for analysis. In both experiments, we checked that excluded participants were not outliers on the symptom questionnaires.

Finally, for each participant, trials with implausibly slow reaction times of >10s and/or ± 3 SD from the per-subject mean were excluded from the analysis. In total, around 1% of all trials were excluded in both experiments.

Drift-Diffusion Model

Decision formation was modelled using the drift-diffusion model (DDM). The DDM models two-choice decision-making as a process of accumulating noisy evidence over time with a certain speed, or drift rate (v). This process terminates when one of two boundaries are crossed, and the associated response is executed. Larger boundary separations (a) lead to slower and more accurate responding. We employed Bayesian estimation of DDM parameters for each subject using the HDDM toolbox (20); (http://ski.clps.brown.edu/hddm_docs/). To ensure each subject's parameter estimates were independent, thus permitting subsequent regression analysis with psychiatric and age/IQ data, each subject's data were fit separately rather than within a hierarchical model. We implemented a regression model to allow the dots difference on each trial, d, to influence the drift rate as follows:

$$v = v_0 + v_\delta * \delta$$

In this equation, the coefficient v_δ (labelled "drift rate (dots difference)" in Figures) encodes the effect of dots difference on drift rate, such that easier trials are associated with greater drifts, and v_0 (labelled "drift rate (baseline)") is an intercept term. The model was fit to accuracy-coded data (i.e., the upper and lower bounds correspond to correct and error responses, and the starting point was fixed at $a/2$) with 4 free parameters: non-decision time, decision threshold, baseline drift rate (v_0) and the effect of dots difference on drift rate (v_δ). For each subject's data, we used Markov chain Monte-Carlo (MCMC) sampling methods to approximate the posterior distributions of the estimated parameters. 3 chains were run each with 2000 samples, with the first 500 samples discarded as burn-in. Convergence was assessed by computing Gelman and Rubin's potential scale reduction statistic $\hat{R}$ for each parameter (21). Large values of $\hat{R}$ indicate convergence problems and values ~1 suggest convergence. The range of $\hat{R}$ values across all subjects and parameter estimates was 0.99-1.01 for Experiment 1 and 0.99-1.07 for Experiment 2, indicating satisfactory convergence. Mean posterior estimates were extracted for entry into subsequent regression analyses. The four DDM parameters were relatively uncorrelated across individuals (Figure S4F).

To determine the ability of the model to reproduce performance outputs that are qualitatively similar to empirical data, we simulated synthetic response times and choices from the DDM at each of the 70 dots difference values present in Experiment 1 using fitted parameters for each subject. The *simuldiff* function from the DMAT toolbox ((22) http://ppw.kuleuven.be/okp/software/dmat/) was used to specify DDM simulations in MATLAB. We simulated 1000 trials for each subject at each dots difference, and calculated summary statistics for both accuracy and response time for the data and model simulations collapsed into 6 difficulty bins (Figures 1B and 1C).

Linear Regressions

We conducted linear regressions to examine the relationship between psychiatric symptoms/age/IQ and task-related variables. For both Experiments 1 and 2, dependent variables characterizing decision formation included *accuracy* (as measured by the slope of the psychometric function in Experiment 1 and mean accuracy in Experiment 2), *non-decision time*, *drift rate (baseline)*, *drift rate (dots difference)*, and *decision threshold*. In Experiment 1, dependent variables characterizing metacognitive evaluation included *confidence level* (i.e. mean trial-by-trial confidence), *global pre-task confidence*, *global post-task confidence*, *global signed update* (*global post-task confidence* - *global pre-task confidence*) and *global absolute update* (|*global post-task confidence* - *global pre-task confidence*|) (Figure S3). In Experiment 2, we controlled task performance to enable measurement of confidence level and metacognitive efficiency (see below), and global ratings were omitted. In both Experiments 1 and 2, we included the total score for each psychiatric questionnaire (log-transformed) and age, IQ and gender as fixed effects. All regressors were z-scored to ensure comparability of regression coefficients. Due to high correlations across the different psychiatric questionnaires, including all the questionnaires scores in the same regression would not produce an interpretable result, such that meaningful shared variance would be lost. Therefore, the associations between each dependent variable and each symptom were first assessed using separate regressions for each symptom, while controlling for age, IQ and gender. In the syntax of the *lm* function in R, the regressions were:

Dependent Variable ~ Symptom + Age + IQ + Gender

In addition, in order to assess the sole influence of the covariates of age, IQ and gender regardless of psychiatric symptoms, we examined how each dependent variable was associated with the age and IQ measures:

$$\text{Dependent Variable} \sim \text{Age} + \text{IQ} + \text{Gender}$$

We applied Bonferroni correction for multiple comparisons over the number of dependent variables tested. All reported statistics are corrected unless otherwise specified. Contrast values were estimated using the *esticon* package in R.

Quantifying Confidence Level (Bias) and Metacognitive Efficiency

In Experiment 2, decision performance was controlled permitting isolation of confidence level and metacognitive efficiency from fluctuations in performance using signal detection theory metrics (see Methods). We characterized the sensitivity of an observer's confidence reports to correct or incorrect judgments using the meta-*d'* statistic (this is known as "type 2" SDT as compared to classic type 1 SDT in which the observer is discriminating a state of the external environment (23)). Meta-*d'* was fit to confidence rating data using maximum likelihood methods implemented in freely available MATLAB code (http://www.columbia.edu/~bsm2105/type2sdt/). We quantified confidence level as the mean trial-by-trial confidence rating, as in Experiment 1. One subject with negative *meta-d'/d'* was removed, leaving 496 subjects for regressions on metacognitive efficiency.

Factor Analysis

In Experiment 2, we used a factor analysis with Maximum Likelihood Estimation to account for the partial overlap between the various questionnaire scores, and explore a more parsimonious latent structure for explaining variation in questionnaire item-level scores. Factor analysis was conducted using the *fa()* function from the Psych package in R, with an oblique rotation (oblimin) as we have used previously (26). 209 individual questionnaire items were entered as measured variables into the factor analysis (Figures 3 and S2). For the social anxiety questionnaire (LSAS), the average of the avoidance and fear/anxiety answers of each item was taken. As responses on the schizotypy scale

were binary at the item-level, a heterogeneous correlation matrix was computed using the *hector* function in *polycor* package in R. This allowed for Pearson correlations between numeric variables, polyserial correlations between numeric and binary items and polychoric correlations between binary variables. We selected the number of factors based on Cattell's criterion (27), in which a sharp "elbow" indicates the point at which there is little benefit to retaining additional factors (Figure 3B). This test was implemented using the Cattell-Nelson-Gorsuch (CNG) test using the nFactors package in R.

Importantly, we replicate previous results using this questionnaire set (26) despite employing a lower subject-to-variable ratio: a 3-factor latent structure was found to best and most parsimoniously explain the item-level responses (Figure 3B), and the loadings across items were highly correlated across the two studies (Figure S9). We employed the same labels as in Gillan et al. (2016), according to the strongest individual items loadings (≥0.25) (Figure 3C and S2B). Factor 1 'Anxious-Depression' was dominated by items from the Generalised Anxiety, Depression and Apathy questionnaires, as well as items from the Impulsivity questionnaire. For Factor 2 'Compulsive Behavior and Intrusive Thought', the highest loadings came from the OCD and Schizotypy questionnaires, as well as items from the Eating Disorders and Alcoholism questionnaires. Lastly, Factor 3 'Social Withdrawal' had the highest average loadings from the Social Anxiety questionnaire, without significant contribution from Generalised Anxiety questionnaire.

As for the individual questionnaire scores, we tested the extent to which the three psychiatric factors were related to decision and metacognitive dependent variables, while controlling for age, IQ and gender:

Dependent variable ~ Factor1 'Anxious-Depression' + Factor2 'Compulsive Behavior and Intrusive Thought' + Factor3 'Social Withdrawal' + Age + IQ + Gender.

Model Comparison

To quantify the extent to which including psychiatric factors in addition to age, IQ and gender explains individual differences in decision formation and metacognitive evaluation we performed a

Bayesian model comparison. We estimated three models for each dependent variable: a null (intercept-only) model (gender alone); a model including only age, gender and IQ and a model including age, gender, IQ and the three psychiatric factor scores. For each regression model, we computed the Bayesian Information Criterion (BIC), which accounts both for likelihood (model evidence) and model complexity (28). Differences in BIC scores were quantified as Null Model – Age/IQ Model (Figure S6C), and Age/IQ Model – Psychiatric Factors Model (Figure 5). The strength of evidence for one model over the other can be inferred from BIC scores as follows: weak for a difference between 0 and 2, positive between 2 and 6; strong between 6 and 10; and very strong for a difference higher than 10 (29).

Supervised Regression Analysis

In addition to the factor analysis, we carried out a supervised regression analysis to identify the individual questionnaires items that explained the most independent variance in confidence level, using linear regression with elastic net regularization (30). Besides minimizing ordinary sum of squared residuals of the regression, elastic net regularization imposes two extra-penalty terms (on the absolute L1 norm and the squared values of the regression coefficients (L2 norm)), allowing selection of a solution with particular properties. For high-dimensional problems, this avoids over- or under-fitting, thus enhancing the accuracy of the predictors, through automatic variable selection and shrinkage of large regression coefficients. All 209 questionnaire item scores and the dependent variable (confidence level) were first scaled using a z-score transform. As in Gillan et al. (2016), we implemented ten-fold cross-validation with nested cross-validation for tuning and validating the model. The data were randomly split into 10 groups. A model was then generated based on 9 training groups, and applied to the remaining independent testing group. Each group served as the testing group once, resulting in 10 different models and predictions based on independent data. Nested cross-validation involved subdividing the 9 training groups (i.e., 90% of the sample) into a further 10 groups ("inner" folds). Within these 10 inner folds, 9 were utilized for training a model over a range of 50 alpha (0.01–1) and 50 lambda (0.001–1) values, where alpha is the complexity parameter and lambda is the regularization coefficient. This generated a model fit on the inner fold test set for each

possible combination of alpha and lambda. The best fit over all 10 inner folds for each combination of alpha and lambda was then used to determine the optimal parameters for each outer fold. We conducted 100 iterations of regularization with tenfold validation and retained items that were significant predictors of confidence level in ≥95% of final models. All tested models were significant, with the median cross-validated ρ=0.27, median cross-validated p<0.000001. 26 out of 209 questionnaire items that met this criterion are listed in Table S1.

Supplemental Figures and Table

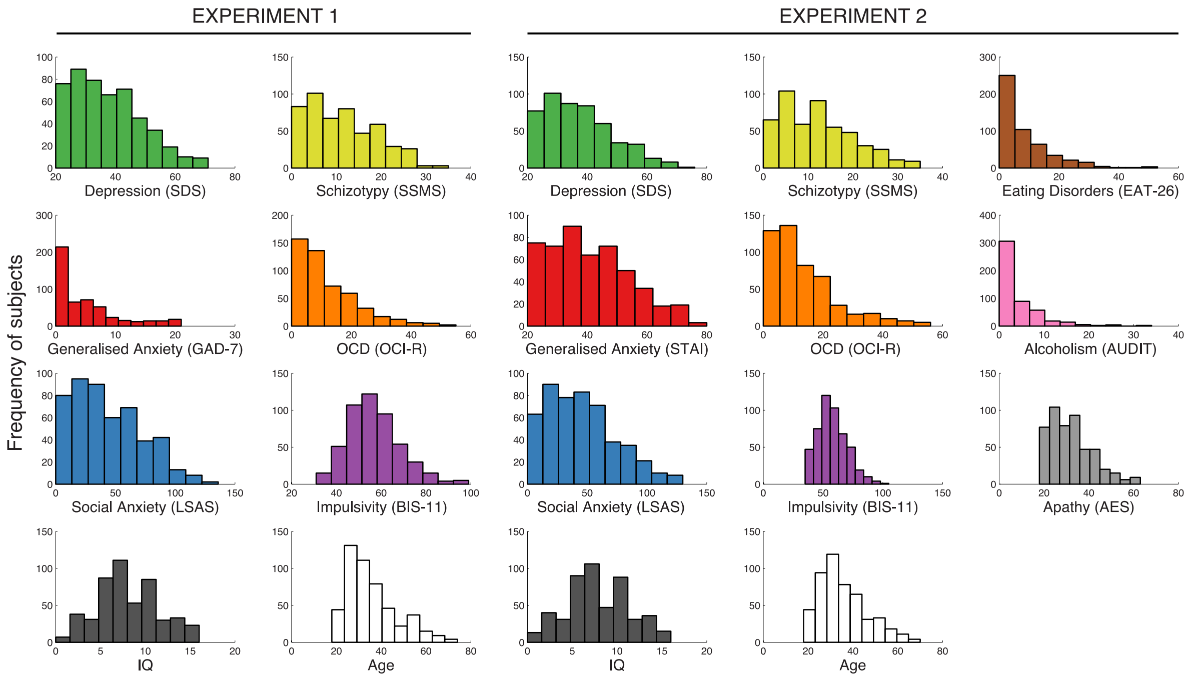

Figure S1 (related to Figure 3). Age, IQ (ICAR score) and psychiatric questionnaire score distributions across participants (Experiment 1: N=498, Experiment 2: N=497).

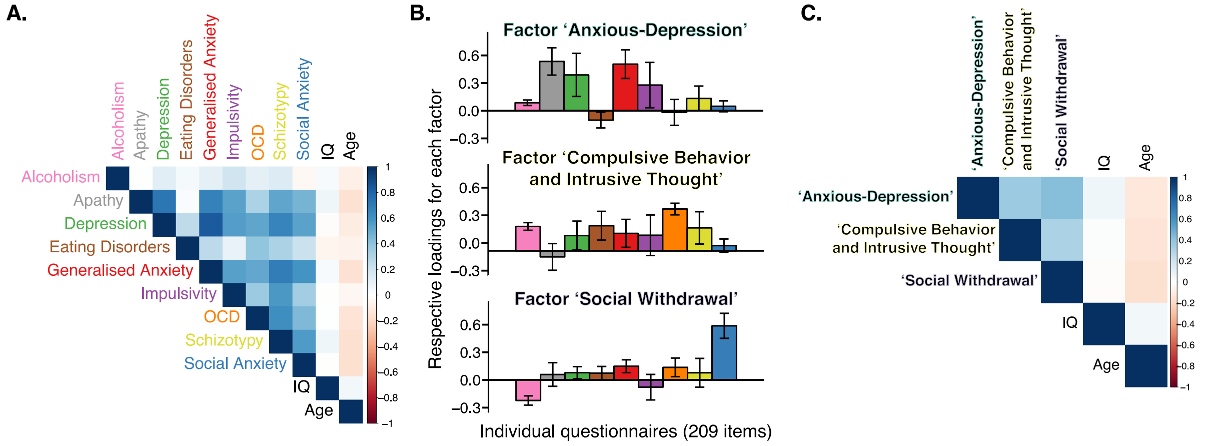

Figure S2 (related to Figure 3). Three latent factors explained the shared variance between all questionnaire items

(A) Correlation matrix of the mean scores of the 9 questionnaires used.

(B) Loadings of mean questionnaire scores onto the 3 latent factors. Error bars denote standard deviation of the mean over item loadings.

(C) Correlation matrix of the three psychiatric factor scores, age and IQ.

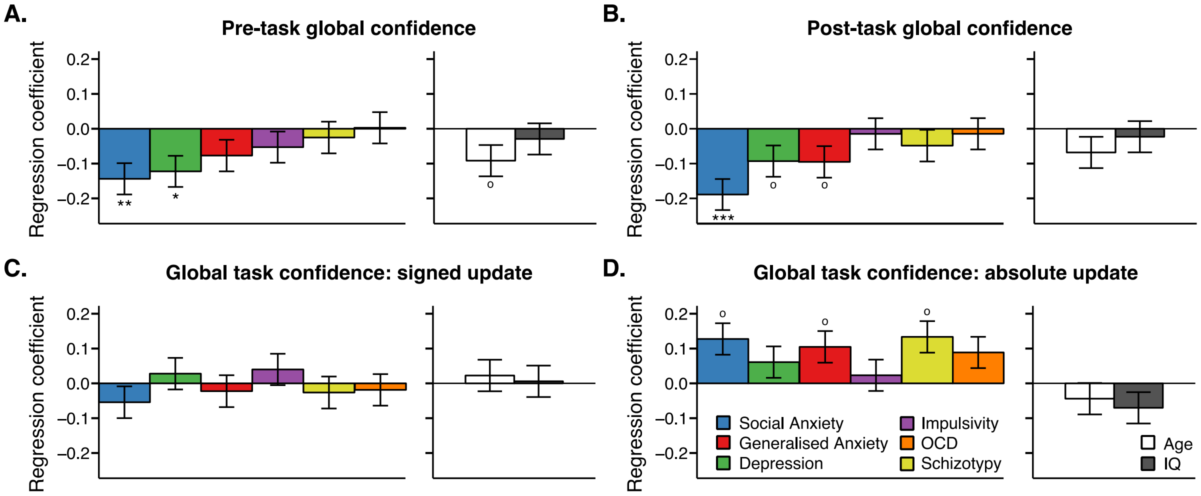

Figure S3 (related to Figure 2). Relationship between psychiatric symptoms, age, IQ and (A) pre-task global confidence rating, (B) post-task global confidence rating, (C) global confidence signed update (post-pre) and (D) global confidence absolute update (Experiment 1). Lower global confidence relative to others was associated with negative affect symptoms, in particular social anxiety. Y-axes indicate the change in each dependent variable for each change of 1 standard deviation of psychiatric symptoms. Error bars denote standard errors. °*p*<.05 uncorrected, **p*<.05, ***p*<.01, ****p*<.001 corrected for multiple comparisons over the number of dependent variables tested.

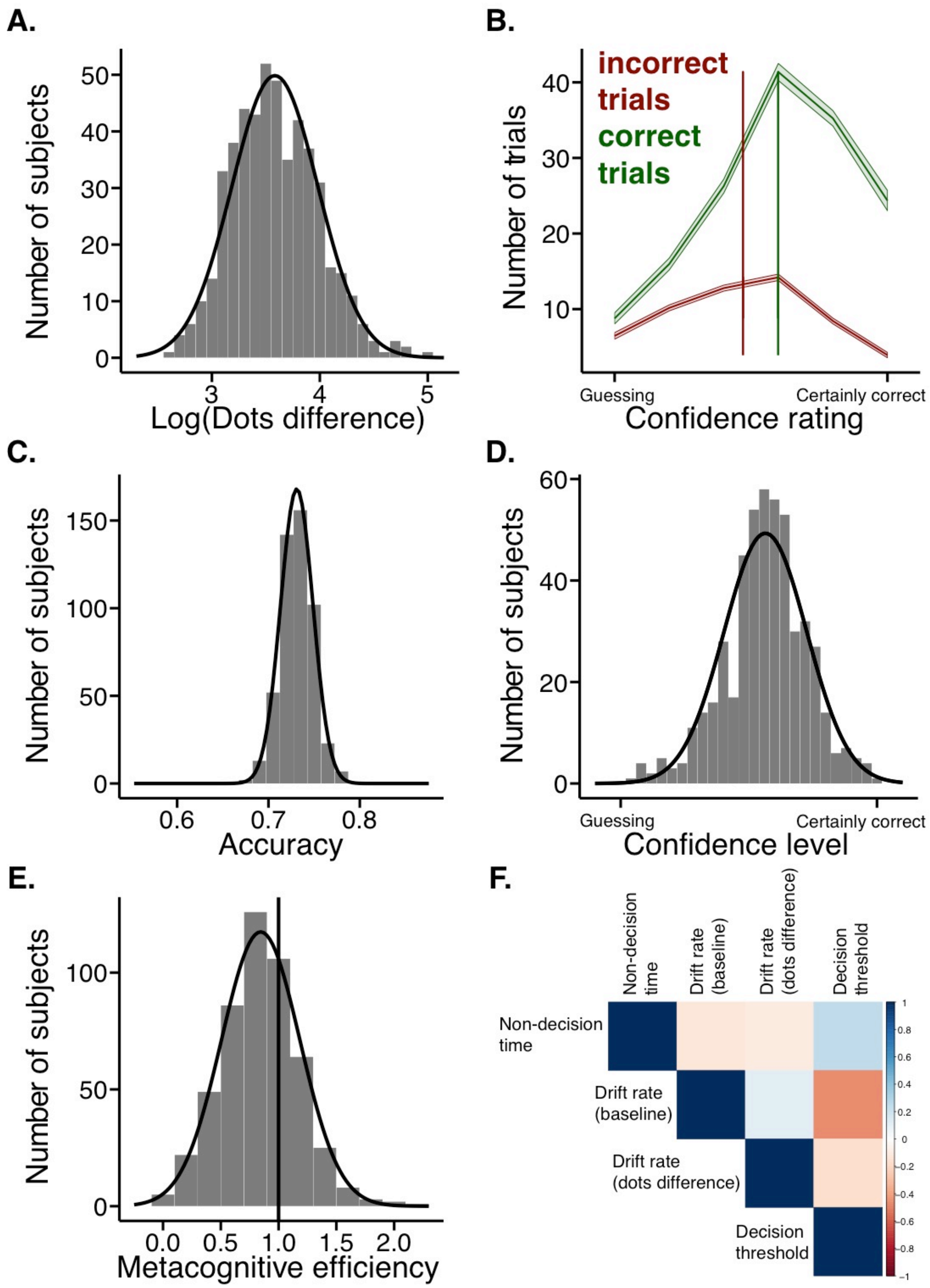

Figure S4. Decision formation and metacognition in Experiment 2 (N=497)
(A) Histogram of average(log(dots difference)) achieved by staircase adjustment across subjects.
(B) Mean confidence for correct (green) and incorrect trials (red). Shaded areas denote S.E.M.
(C, D, E) Distribution of (C) mean accuracy, (D) mean confidence level and (E) metacognitive efficiency across subjects.
(F) Correlation matrix of drift-diffusion model fitted parameters.

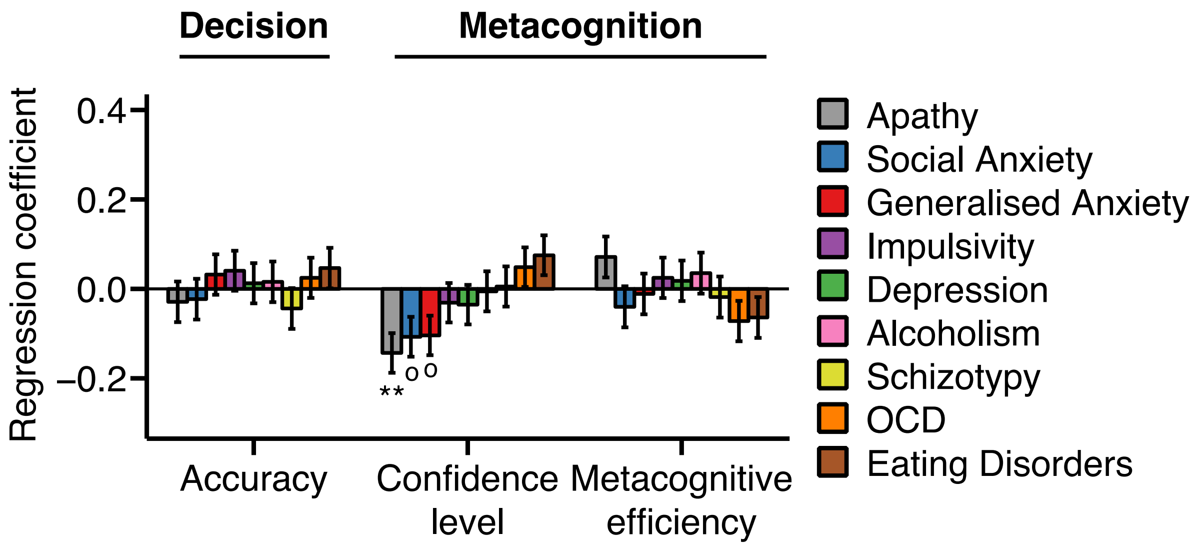

Figure S5. Associations between decision and metacognitive variables and self-reported psychiatric symptoms in Experiment 2. The Y-axis indicates the change in each dependent variable for each change of 1 standard deviation of symptoms. Replicating results from Experiment 1, greater levels of negative affect symptoms (anxiety and apathy) were associated with lower confidence level, despite no effects on decision accuracy. Error bars denote standard errors. $^{o}p<.05$ uncorrected, $^{**}p<.01$ corrected for multiple comparisons over the number of dependent variables tested.

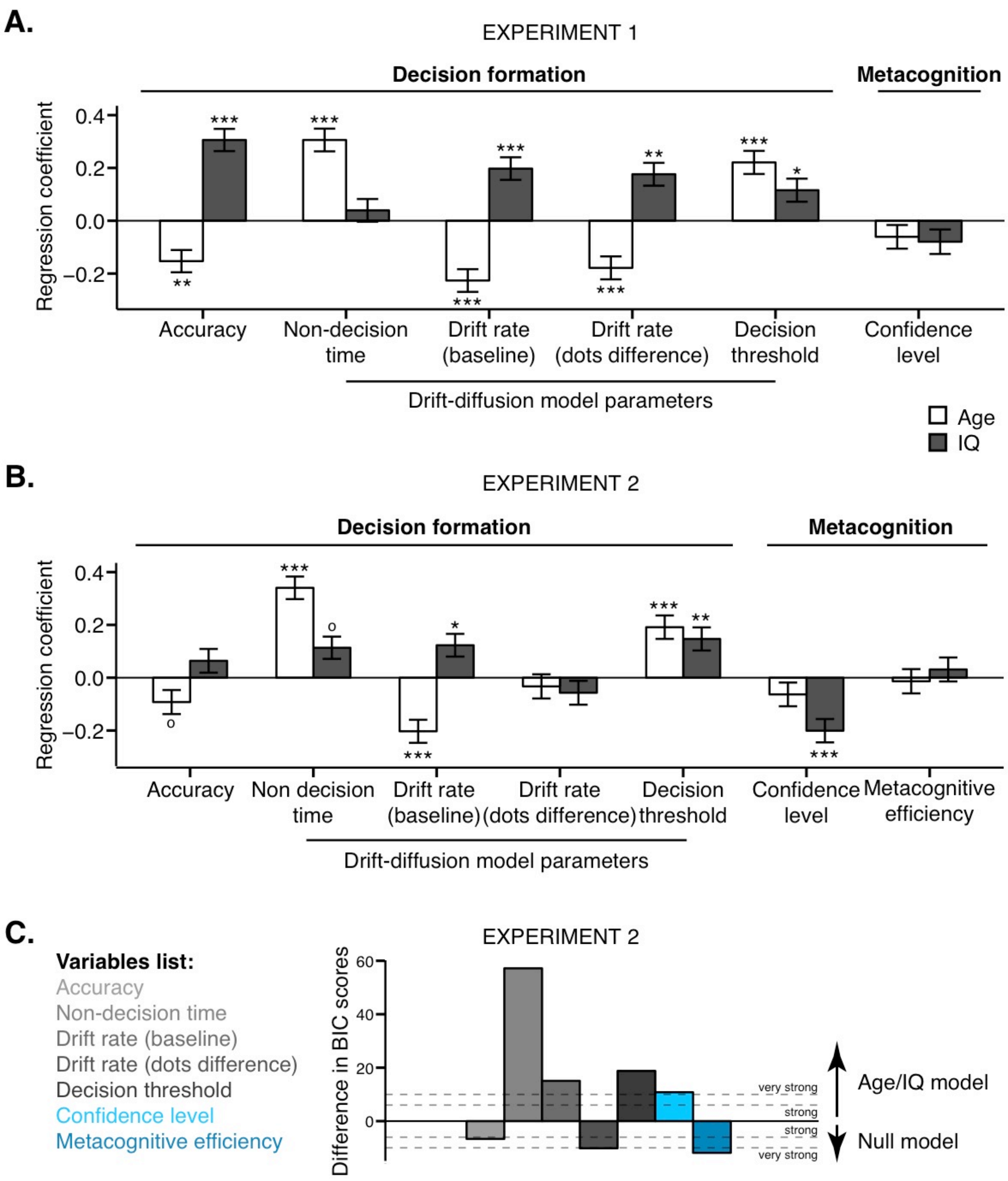

Figure S6. Association between decision formation (left) and metacognitive (right) variables with age/IQ in (A) Experiment 1 (N=498) and (B) Experiment 2 (N=497). Y-axes indicate the change in each dependent variable for each change of 1 standard deviation of age/IQ. In Experiment 1, age and IQ predicted parameters governing decision formation, but not confidence level. In Experiment 2, age and IQ also predicted parameters governing decision formation, although here we additionally observed a negative relationship between confidence level and IQ. Error bars denote standard errors. $^{o}p<.05$ uncorrected, $^{*}p<.05$, $^{**}p<.01$, $^{***}p<.001$ corrected for multiple comparisons over the number of dependent variables tested.

(C) Model comparison in Experiment 2. Taking into account both goodness of fit and parsimony, model comparison provided strong evidence for including age and IQ for explaining changes in aspects of decision formation and confidence level. Null model: variable ~ Gender. Age/IQ model: variable ~ Age + IQ + Gender. See also Figure 5.

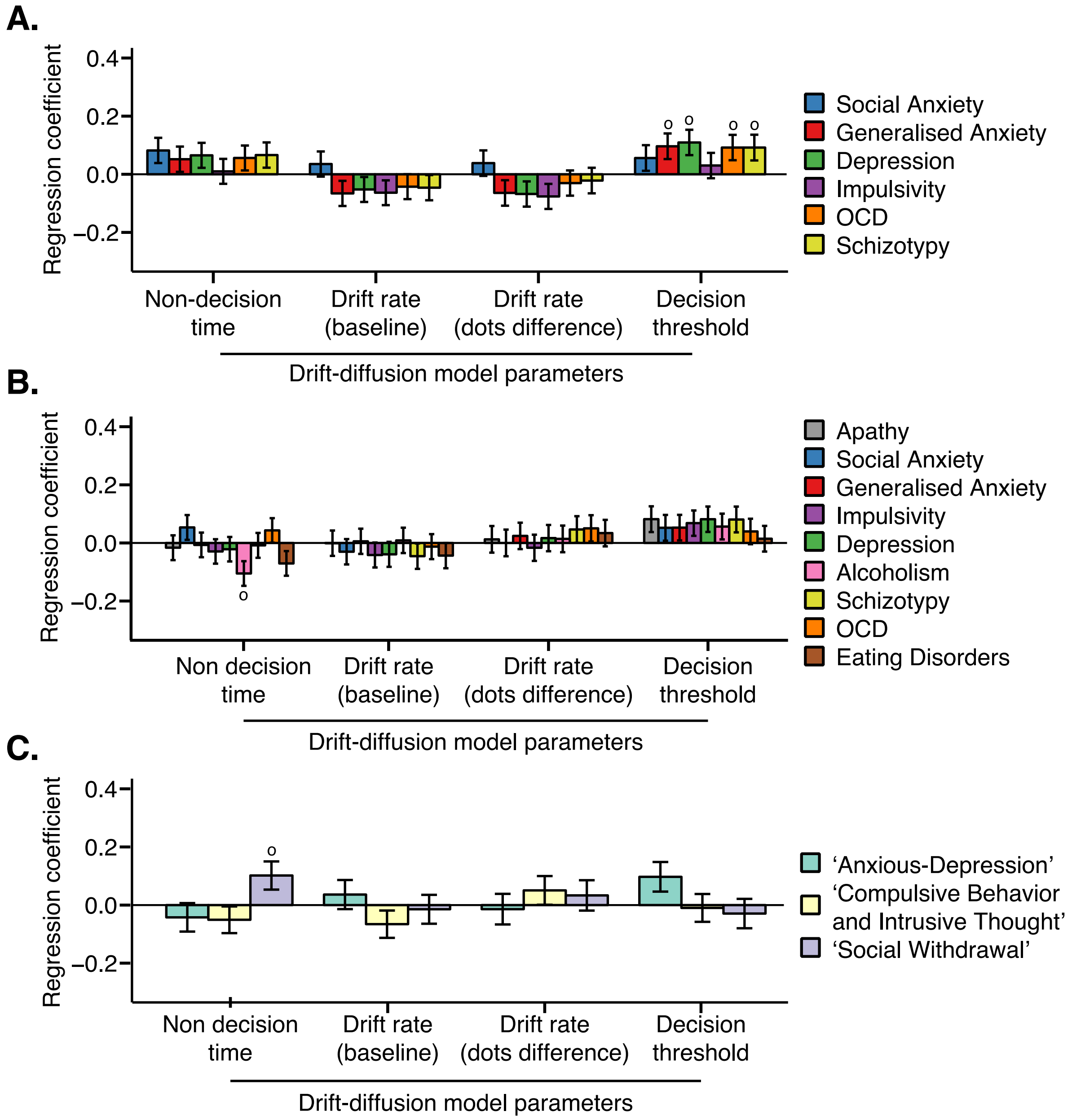

Figure S7. Association between decision formation (i.e. drift-diffusion model parameters) and psychiatric symptoms in (A) Experiment 1, (B) Experiment 2, and (C) psychiatric factors in Experiment 2. No consistent relationship was found between psychiatric symptoms/factors and parameters characterizing decision formation. Y-axes indicate the change in each dependent variable for each change of 1 standard deviation of psychiatric symptoms. Error bars denote standard errors. °*p*<.05 uncorrected.

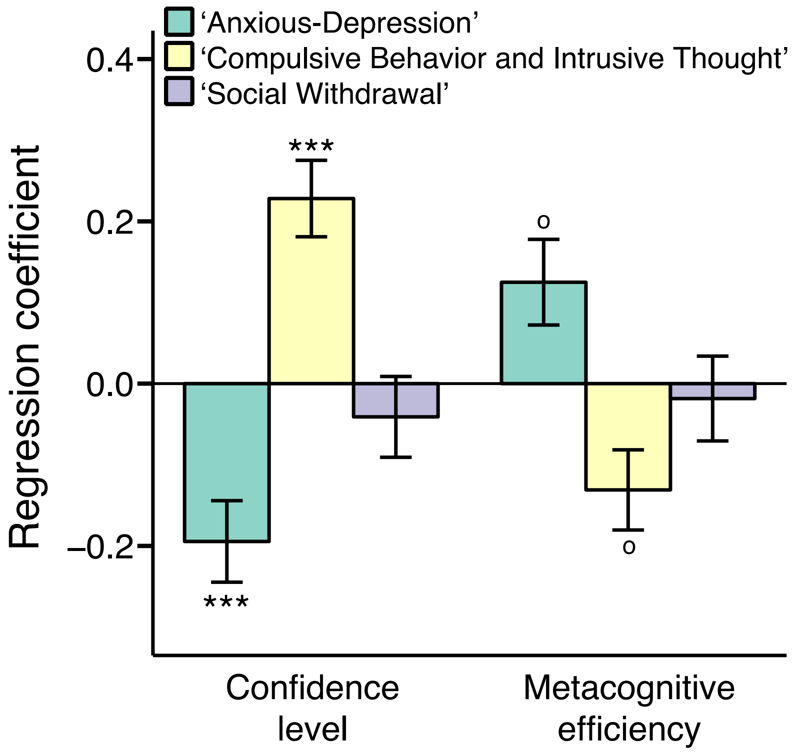

Figure S8 (related to Figure 4). Associations between metacognitive variables and the three symptom dimensions from the factor analysis, controlling for decision formation (accuracy and all DDM parameters), age, IQ and gender. Relationships between psychiatric factors and both confidence level and metacognitive efficiency were maintained when additionally controlling for aspects of decision formation. The Y-axis indicates the change in each dependent variable for each change of 1 standard deviation of psychiatric symptoms. Error bars denote standard errors. °*p*<.05 uncorrected, ****p*<.001 corrected for multiple comparisons over the number of dependent variables tested.

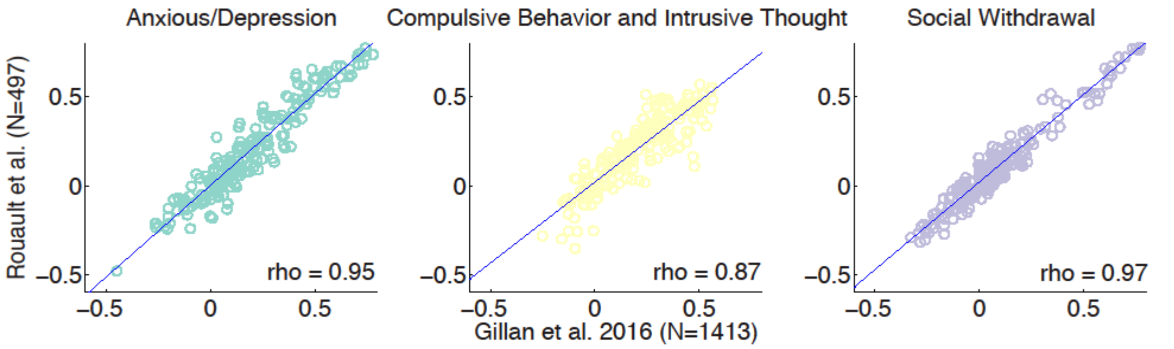

Figure S9 (related to Figure 3). Correlations between item loadings from the factor analysis in Gillan et al., (2016) and the present study for the three symptom dimensions. Questionnaire item loadings were highly correlated indicating recovery of similar symptom dimensions across the two studies.

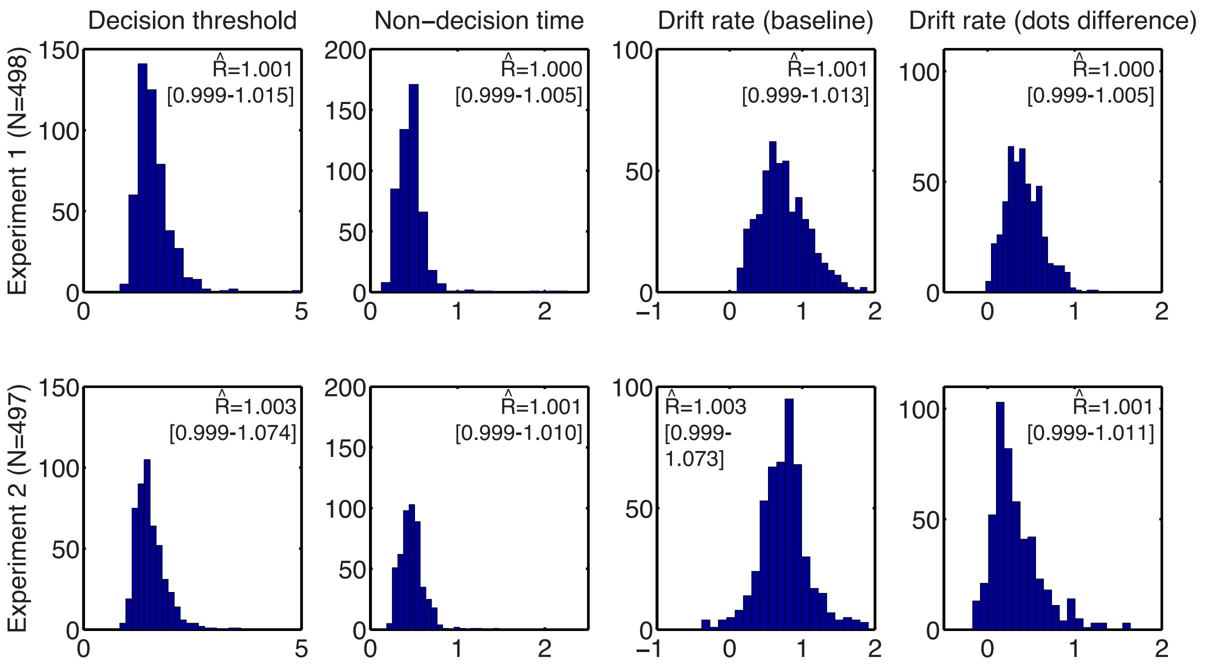

Figure S10: HDDM parameter distributions across subjects for Experiment 1 (N=498, top panels) and Experiment 2 (N=497, bottom panels). Convergence values $\hat{R}$ (mean [minimum-maximum]) are reported for each of the four parameters, indicating good reliability of the fitting procedure.

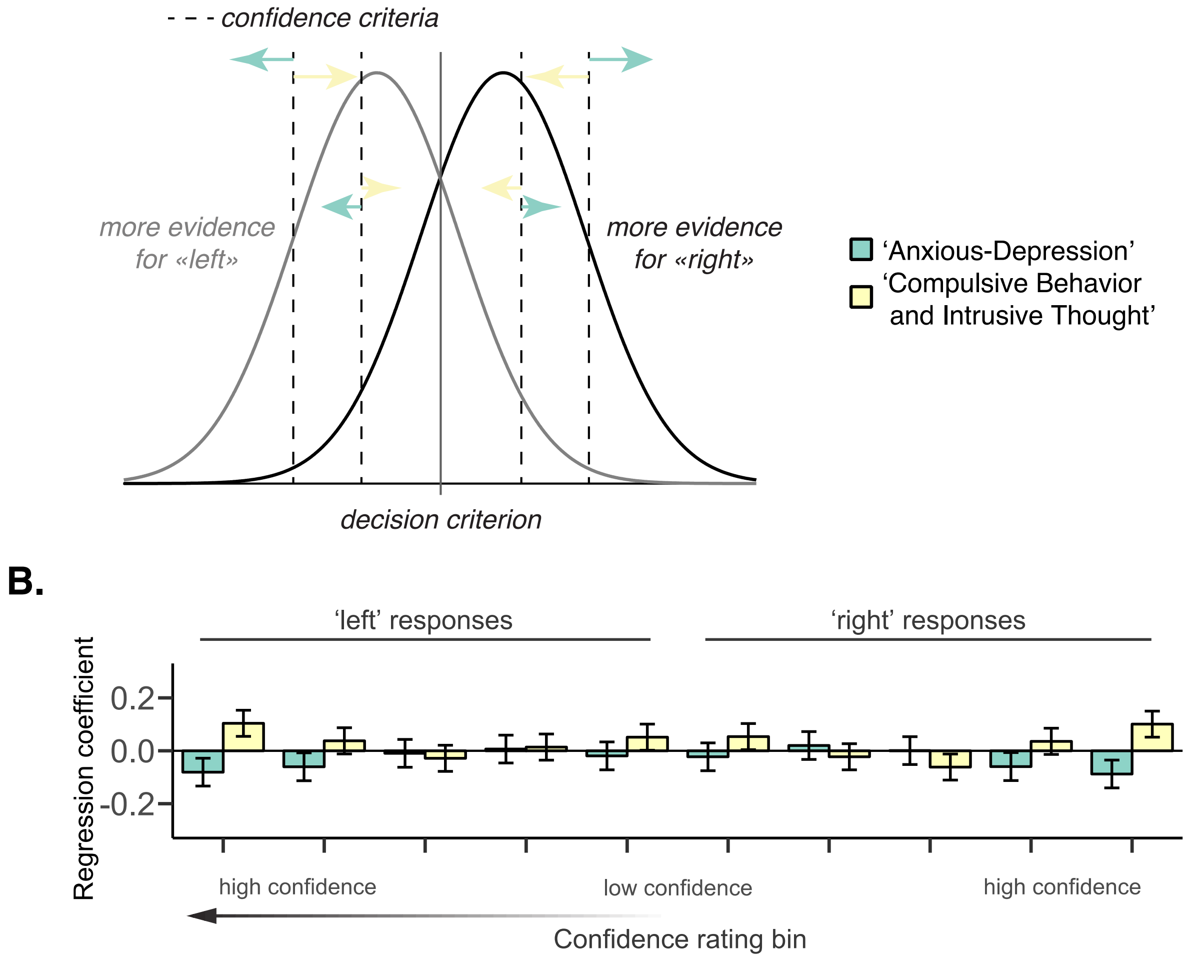

Figure S11.
(A) Signal detection theory model underlying the metacognitive efficiency fits. The flanking confidence criteria (dotted lines) indicate the points at which greater evidence for left/right translates into a higher confidence rating. See also Supplemental Results.
(B) Absolute distances between confidence criteria and decision criterion from the meta-*d'* model fit (divided by meta-*d'*) were regressed against the 'Anxious-Depression' and 'Compulsive Behavior and Intrusive Thought' dimensions. Error bars denote standard errors. Y-axes indicate the change in each dependent variable for each change of 1 standard deviation of psychiatric factor scores.

Table S1. Significant predictors of confidence level from supervised regression analysis in Experiment 2, listed by effect size from negative to positive predictors

Questionnaire	Item	Index	Beta	FA loadings
Generalised Anxiety	I am (not) "calm, cool and collected"	STAI-07	-0.062	+F1
Impulsivity	I (do not) like puzzles	BIS-29	-0.054	-F2
Impulsivity	I change jobs	BIS-16	-0.040	+F1
Apathy	I (do not) spend time doing things that interest me	AET-09	-0.038	+F1
Apathy	I (do not) have friends	AET-12	-0.034	+F1
Social Anxiety	Giving a party (fear and avoidance of)	LSAS-23	-0.030	+F3
Eating Disorders	I take longer than others to eat meals	EAT-15	-0.029	+F2
Schizotypy	Has dancing or the idea of it always seemed dull to you?	SSMS-29	-0.028	-
Eating Disorders	I display self-control around food	EAT-19	-0.025	-
Generalised Anxiety	I (do not) feel pleasant	STAI-01	-0.024	+F1
Schizotypy	Is it hard for you to make decisions?	SSMS-22	-0.024	+F1
Impulsivity	I am (not) future oriented	BIS-30	-0.021	+F1
Apathy	I am (not) interested in learning new things	AET-05	-0.021	+F1 -F2
Impulsivity	I say things without thinking	BIS-14	0.020	+F2
Eating Disorders	I avoid eating when I am hungry	EAT-02	0.021	-
Generalised Anxiety	I get in a state of tension or turmoil as I think over my recent concerns and interests	STAI-20	0.022	+F1 +F2
Schizotypy	Do you often feel like doing the opposite of what other people suggest even though you know they are right?	SSMS-43	0.024	+F2
Schizotypy	Does a passing thought ever seem so real that it frightens you?	SSMS-09	0.028	+F2
Eating Disorders	I like my stomach to be empty	EAT-24	0.031	+F2
Schizotypy	Have you sometimes sensed an evil presence around you, even though you could not see it?	SSMS-04	0.034	+F2
Apathy	Someone has to tell me what to do each day	AET-10	0.038	-
Depression	I have crying spells or feel like it	SDS-03	0.039	+F2
Impulsivity	I make-up my mind quickly	BIS-03	0.041	-
Impulsivity	I am happy-go-lucky	BIS-04	0.044	-F1
Impulsivity	I do things without thinking	BIS-02	0.048	+F1 +F2
Schizotypy	Are there very few things that you ever enjoyed doing?	SSMS-24	0.052	-

Features that are significantly associated with confidence level identified using elastic net regularization with tenfold cross-validation, observed in ≥95% of 100 iterations tested. Index refers to the item number from the questionnaire of origin. Beta refers to the median regression coefficient of all regularized regression models. Words in parentheses, e.g. "(do not)" are added here (but were not presented to participants) to facilitate interpretation of the direction of effects for items that are reverse coded. The last column "FA Loadings" indicates the significant overlap in terms of loading on Factors F1 ('Anxious-Depression'), F2 ('Compulsive Behavior and Intrusive Thought') and/or F3 ('Social Withdrawal'), in the positive (+) or negative (-) direction using a cut-off of loadings ≥0.25 (as in (26)).

Supplemental Results

Global Confidence Ratings (Experiment 1)

We also observed a relationship between psychiatric symptom scores and "global" confidence ratings about overall performance relative to others, estimated before and after performing the task (Figure S3 and Supplemental Methods). Higher social anxiety and depression scores were predictive of lower pre-task (both β<-0.12, p<0.05) and post-task (social anxiety, β=-0.19, p<0.01; depression, β<-0.09, p<0.05 uncorr.) confidence ratings. Together with confidence level, the findings of Experiment 1 suggest that anxious-depression symptoms are associated with specific shifts in metacognition but not performance in perceptual decision-making.

Behavioral Results (Experiment 2)

Average accuracy ranged from 67.46% to 78.47% correct across subjects (mean=73.10%, SD=1.78%), indicating that the two-down one-up staircase adequately controlled for performance (Figure S4C). As in Experiment 1, subjects used the confidence rating scale appropriately, giving higher confidence on correct trials, and lower confidence on incorrect trials (Figure S4B). There was also considerable individual variability in decision performance (Figure S4). The average task difficulty (mean log(dots difference)) ranged from 2.64 to 5.01 (mean=3.58, SD=0.40) (Figure S4A) and mean confidence ranged from 1.21 to 5.91 across subjects (mean=3.83, SD=0.81) (Figure S4D). Metacognitive efficiency i.e. *meta-d'/d'* clustered near 1, the expected value under a signal detection theory model of confidence (mean=0.84, SD=0.34) (Figure S4E). Individually fitted drift-diffusion model parameters were relatively uncorrelated, indicating that they captured non-overlapping features of the decision-making process (Figure S4F).

To assess the relative significance of the relationships between metacognition and symptom dimensions (Figure 4), we next entered metacognitive variables and accuracy as predictors of individual factor scores in separate regressions. Factor scores were significantly explained by confidence level (β=-0.13 for AD and β=0.15 for CIT, both p<0.003) but not accuracy (both p>0.6) or metacognitive efficiency (p=0.1 for AD, trend at p=0.04 for CIT). In addition, the association between

each factor and confidence level effect was greater in magnitude than the corresponding relationship with accuracy (both $p<0.03$).

Relationship Between Decision Formation Parameters and Age/IQ (Experiments 1 and 2)

A canonical finding is that older age leads to slower decisions, through an increase in response caution and greater non-decision time in sequential sampling models (31). We therefore additionally investigated (and controlled for in our psychiatric analyses) the influence of age on decision time. As expected, in Experiment 1 we found that older participants had longer non decision times (β=0.31, $p<0.001$), lower drift rates (baseline β=-0.18, $p<0.001$, dots difference β=-0.23, $p<0.001$), higher decision thresholds (β=0.22, $p<0.001$) and lower decision accuracy (β=-0.15, $p<0.01$) (Figure S6A). Subjects with higher IQ exhibited higher drift rates (baseline β=0.18, $p<0.001$, dots difference β=0.20, $p<0.001$) indicating better integration of evidence, as well as higher decision thresholds (β=0.12, $p<0.05$), which together resulted in higher accuracy (β=0.31, $p<0.001$). Importantly, neither age nor IQ nor gender was related to confidence level (Figure S6A). Taken together, our findings reveal a double dissociation: while age and IQ were significantly associated with decision formation but not confidence, psychiatric symptom scores predicted changes in confidence but not in decision formation.

In Experiment 2, we replicated the relationship between age, IQ and changes in decision formation, although effects on accuracy and drift rate (dots difference coefficient) were no longer significant, presumably due to limited fluctuations in performance (Figure S6B). We also observed a significant relationship between IQ and confidence level (β=-0.20, $p<0.001$), unlike in Experiment 1 (see Supplemental Discussion). Neither confidence level nor metacognitive efficiency was associated with gender. A model comparison indicated very strong evidence in favour of including age and IQ compared to a null model in explaining aspects of decision formation and evaluation, including non-decision time, drift rate (baseline), decision threshold, and confidence level (Figure S6C).

Shifts in Confidence Criteria Under the Meta-*d'* Model (Experiment 2)

Shifts in overall confidence are accommodated in signal detection theory as shifts in the amount of evidence needed to emit a particular confidence rating, modelled as confidence criteria (Figure S11A). When these criteria become more conservative (increased distance from the decision criterion), more evidence is needed before high confidence reports are given. In contrast, when they become more liberal (smaller distance from the decision criterion), less evidence is needed for a high confidence report. To quantify such effects we extracted the fitted confidence criteria from the meta-*d'* model fit (the absolute distances between the decision criterion and the flanking confidence criteria, divided by meta-*d'*) and regressed these onto symptom dimensions (Figure S11B). We observed negative/positive relationships between confidence criteria and AD/CIT symptom scores; however, for both factors, these relationships were more pronounced for more extreme confidence criteria, indicating symptoms were most related to changes in the use of higher confidence ratings (Figure S11).

Supervised Analysis (Experiment 2)

We sought to identify which of the 209 individual questionnaire items were most predictive of changes in confidence level in an independent supervised regression (Supplemental Methods). Consistent with our classical regression analyses, we found that ~2/3 of the items negatively predicting confidence level (e.g. "Is it hard for you to make decisions?") were associated with the 'Anxious-Depression' dimension. In contrast, ~2/3 of the items positively predicting confidence level (e.g. "Do you often feel like doing the opposite of what other people suggest even though you know they are right?") overlapped with the 'Compulsive Behavior and Intrusive Thought' dimension (Table S1).

Supplemental Discussion

We found that age modulated decision formation parameters, replicating previous results linking aging to slower decisions, as manifest by alterations in evidence accumulation (31; 32). These findings reveal a robust double dissociation – while psychiatric symptoms predicted changes in metacognition but not decision performance, age predicted changes in decision performance but not metacognition. Other work suggests that metacognitive efficiency decreases in older age (33), but this change was found to be specific to a visual contrast detection task. It remains to be seen whether the link between confidence, age and psychopathology generalises to other tasks or domains. We also examined the influence of IQ on decision formation processes; namely, higher IQ was associated with better evidence accumulation as measured by higher drift rates, indicating a better quality of integrated information (31). In contrast, we observed an inconsistent relationship between IQ and confidence: IQ did not predict confidence level in Experiment 1, whereas a negative relationship was obtained in Experiment 2. It remains possible that changes in calibration and/or confidence reporting requirements in Experiment 2 unmasked a latent association between IQ and confidence level, but further work is required to tease apart these possibilities.

Supplemental References

1. Fleming SM, Ryu J, Golfinos JG, Blackmon KE (2014): Domain-specific impairment in metacognitive accuracy following anterior prefrontal lesions. *Brain*. 137: 2811–2822.

2. Boldt A, Yeung N (2015): Shared neural markers of decision confidence and error detection. *J Neurosci*. 35: 3478–3484.

3. De Leeuw JR (2015): jsPsych: A JavaScript library for creating behavioral experiments in a Web browser. *Behavior Research Methods*. 47: 1–12.

4. Fleming SM, Weil RS, Nagy Z, Dolan RJ, Rees G (2010): Relating introspective accuracy to individual differences in brain structure. *Science*. 329: 1541–1543.

5. García-Pérez MA (1998): Forced-choice staircases with fixed step sizes: asymptotic and small-sample properties. *Vision Research*. 38: 1861–1881.

6. Zung WW (1965): A Self-Rating Depression Scale. *Archives of general psychiatry*. 12: 63.

7. Spitzer RL, Kroenke K, Williams JB, Löwe B (2006): A brief measure for assessing generalized anxiety disorder: the GAD-7. *Archives of internal medicine*. 166: 1092–1097.

8. Mason O, Linney Y, Claridge G (2005): Short scales for measuring schizotypy. *Schizophrenia research*. 78: 293–296.

9. Patton JH, Stanford MS (1995): Factor structure of the Barratt impulsiveness scale. *Journal of clinical psychology*. 51: 768–774.

10. Foa EB, Huppert JD, Leiberg S, Langner R, Kichic R, Hajcak G, Salkovskis PM (2002): The Obsessive-Compulsive Inventory: development and validation of a short version. *Psychological assessment*. 14: 485.

11. Liebowitz MR (1987): *Social phobia*. Karger Publishers.

12. Condon DM, Revelle W (2014): The International Cognitive Ability Resource: Development and initial validation of a public-domain measure. *Intelligence*. 43: 52–64.

13. Spielberger CD, Gorsuch RL, Lushene RD, Vagg PR, Jacobs GA (1983): Manual for the state-trait anxiety inventory (STAI) Consulting Psychologists Press: Palo Alto. *CA, USA*.

14. Saunders JB, Aasland OG, Babor TF, la Fuente De JR, Grant M (1993): Development of the alcohol use disorders identification test (AUDIT): WHO collaborative project on early detection of persons with harmful alcohol consumption-II. *Addiction*. 88: 791–804.

15. Marin RS, Biedrzycki RC, Firinciogullari S (1991): Reliability and validity of the Apathy Evaluation Scale. *Psychiatry research*. 38: 143–162.

16. Garner DM, Olmsted MP, Bohr Y, Garfinkel PE (1982): The eating attitudes test: psychometric features and clinical correlates. *Psychol Med*. 12: 871–878.

17. Button KS, Ioannidis JP, Mokrysz C, Nosek BA, Flint J, Robinson ES, Munafò MR (2013): Power failure: why small sample size undermines the reliability of neuroscience. *Nat Rev Neurosci*. 14: 365–376.

18. Oppenheimer DM, Meyvis T, Davidenko N (2009): Instructional manipulation checks: Detecting satisficing to increase statistical power. *Journal of Experimental Social Psychology*. 45: 867–872.

19. Chandler J, Mueller P, Paolacci G (2014): Nonnaïveté among Amazon Mechanical Turk workers: Consequences and solutions for behavioral researchers. *Behavior Research Methods*. 46: 112–130.

20. Wiecki TV, Sofer I, Frank MJ (2013): HDDM: hierarchical bayesian estimation of the drift-diffusion model in python. *Frontiers in neuroinformatics*. 7: 14.

21. Gelman A, Rubin DB (1992): Inference from Iterative Simulation Using Multiple Sequences. *Statistical Science*. 7: 457–511.

22. Vandekerckhove J, Tuerlinckx F (2008): Diffusion model analysis with MATLAB: A DMAT primer. *Behavior Research Methods*. 40: 61–72.

23. Clarke FR, Birdsall TG, Tanner WP Jr (1959): Two types of ROC curves and definitions of parameters. *The Journal of the Acoustical Society of America*. 31: 629–630.

24. Maniscalco B, Lau H (2012): A signal detection theoretic approach for estimating metacognitive sensitivity from confidence ratings. *Consciousness and Cognition*. 21: 422–430.

25. Fleming SM, Lau H (2014): How to measure metacognition. *Frontiers in human neuroscience*. 8: 1–9.

26. Gillan CM, Kosinski M, Whelan R, Phelps EA, Daw ND (2016): Characterizing a psychiatric symptom dimension related to deficits in goal-directed control. *eLife*. 5: e11305.

27. Cattell RB (1966): The scree test for the number of factors. *Multivariate behavioral research*. 1: 245–276.

28. Schwarz G (1978): Estimating the dimension of a model. *The annals of statistics*. 6: 461–464.

29. Kass RE, Raftery AE (1995): Bayes factors. *Journal of the American Statistical Association*. 90: 773–795.

30. Zou H, Hastie T (2005): Regularization and variable selection via the elastic net. *Journal of the Royal Statistical Society: Series B (Statistical Methodology)*. 67: 301–320.

31. Ratcliff R, Thapar A, McKoon G (2010): Individual differences, aging, and IQ in two-choice tasks. *Cognitive Psychology*. 60: 127–157.

32. Dutilh G, Forstmann BU, Vandekerckhove J, Wagenmakers E-J (2013): A diffusion model account of age differences in posterror slowing. *Psychology and Aging*. 28: 64–76.

33. Palmer EC, David AS, Fleming SM (2014): Effects of age on metacognitive efficiency. *Consciousness and Cognition*. 28: 151–160.
